# Response to letter

**DOI:** 10.1590/0037-8682-0406-2020

**Published:** 2020-11-13

**Authors:** Raghunandanan Rama Varma, Parvathi Varma, Anil Kumar

**Affiliations:** 1 Krishna Hospital, Consultant Ophthalmologist, Kochi, Kerala, India.; 2 Amrita Institute of Medical Sciences, Molecular Biology, Research Scientist, Amrita Vishwa Vidyapeetham, Ponekara, Kochi, Kerala, India.; 3 Amrita Institute of Medical Sciences, Department of Microbiology, Amrita Vishwa Vidyapeetham, Ponekara, Kochi, Kerala India.

The authors thank Prof. *Filipe Dantas-Torres* for his insightful letter on our article “Tick infestation of the eyelid,” which has recently been published in the *Revista Brasileira de Medicina Tropical* ^1^. We agree with all the shortcomings pointed out by Prof. Filipe with all humility.

We feel that the reason for these glaring inconsistences is the fact that we, the authors, are clinicians and microbiologists and do not have the expertise of an entomologist of Prof. Filipe’s caliber. The section “Images in Infectious Diseases” has a word limit of 250 words; therefore, it was not possible to explain the entomology of ticks. With regard to the quality of images, the reviewers and the editorial board were satisfied with the images we had submitted. It should also be noted that the images were captured in the clinic with whatever means available at that time. The intent of the article was to bring awareness among clinicians and clinical microbiologists about this unique condition of tick infestation and possible zoonotic diseases associated with it. The absence of evidence of a pathogen like *Rickettsia rickettsii* cannot be taken as the evidence of its absence in that geographic location (like India).

“The eyes do not see what the mind does not know.” It was a humble effort from the authors to let the readers of your prestigious journal learn about this entity. The scope of the journal *Revista Brasileira de Medicina Tropical* as per the website is tropical medicine (including epidemiology, clinical studies, pathology, immunology, and infectious diseases) and not tropical parasitology. Therefore, our article is well within the scope of the journal. The authors also feel that some of the critiques would have been justified had the article been submitted to vector-specific journals like *Ticks and Tick-borne Diseases* and *Parasites and Vectors*. Once again, we thank Prof. Filipe Dantas-Torres for enriching our knowledge about the intricacies in identifying ticks and associated diseases.
